# Exposure Assessment of Essential and Potentially Toxic Elements (PTEs) from Chia Seeds

**DOI:** 10.3390/jox14040098

**Published:** 2024-11-28

**Authors:** Dailos González-Weller, Elena Bethencourt-Barbuzano, Katarzyna Siedzik, Soraya Paz-Montelongo, Ángel J. Gutiérrez-Fernández, Arturo Hardisson, Samuel Alejandro-Vega, Juan R. Jáudenes-Marrero, Carmen Rubio

**Affiliations:** 1 Grupo de Investigación en Toxicología Ambiental y Seguridad de los Alimentos y Medicamentos, Universidad de La Laguna, 38071 La Laguna, Canary Islands, Spain; dgonzal@ull.edu.es (D.G.-W.); spazmont@ull.edu.es (S.P.-M.); ajguti@ull.edu.es (Á.J.G.-F.); atorre@ull.edu.es (A.H.); salejand@ull.edu.es (S.A.-V.); 2 Servicio de Inspección y Laboratorio, Area de Salud de Tenerife, Gobierno de Canarias, 38006 Santa Cruz de Tenerife, Canary Islands, Spain; 3 Doctoral School, Poznan University of Physical Education, 61-871 Poznan, Poland

**Keywords:** chia seeds, essential elements, exposure assessment, potentially toxic elements, risk characterization

## Abstract

The increasing consumption of chia seeds is followed by a growing interest in their nutritional and toxicological characterization. To assess the characterization of the essential and PTEs of this novel food, 20 samples of conventional and organic chia seeds available on the European market were analyzed using ICP-OES. Then, the dietary exposure to these elements was assessed. An exhaustive investigation into the metal content of this food serves to elucidate the paucity of existing knowledge. The results show that the levels of essential elements are similar in both types of production, while the levels of PTEs are higher in the organic samples. The exposure assessment indicates that Mn contributes the most among the essential elements, followed by Cu in women. Exposure to PTEs through doses of 50 g/day of chia seeds analyzed would hardly pose short-term health risks as the contributions are below 10%, except for Sr, although they could produce a long-term toxicological risk. To promote safety in chia seed consumption, it is recommended to encourage responsible and moderate consumption, continue monitoring PTE levels in this novel food, and establish concentration limits for PTEs in future European regulations.

## 1. Introduction

Chia seeds began to be marketed in Europe in 2009 after their authorization. Since then, their consumption has been growing exponentially and, in 2018, Germany was the main consumer and importer of this product, followed by the Netherlands [1,2,3]. In 2022, the global market for these products was estimated at USD 360.14 million but is expected to increase to USD 626.47 million by 2029 [2].

Chia seeds are an important source of protein for those following vegan diets since they provide all the essential amino acids [1]. Likewise, consumers who require an extra supply of proteins, such as athletes, have found these seeds to be an excellent ingredient to include in their diet. To respond to the growing demand for this novel food, the food industry has strategically included chia seeds in a wide variety of foods such as baked goods, cereals, salads, yogurts, ready meals, milk drinks, and fruit smoothies [1,4,5,6].

Chia (*Savia hispanica*) is an annual plant that belongs to the *Lamiaceae* family and has been widely known since ancient times for its medicinal and dietary properties [7,8]. Its small-sized seeds of different colors are mainly made up of fiber, proteins, essential fatty acids, and antioxidants [9,10]. Furthermore, chia seeds are known for their content of a broad spectrum of tocopherols, phytosterols, carotenoids, flavonoids, and polyphenolic compounds [1], and high amounts of vitamins and minerals such as Ca, P, and Zn [10]. Because of this wide and nutritionally interesting composition, chia seeds have been associated with beneficial effects in relation to aging, cardiovascular and degenerative diseases, obesity, diabetes, and some types of cancer such as liver, pancreatic, colon, and breast cancer [11,12,13,14]. High consumption of chia seeds has been associated with a decrease in triglycerides and blood pressure, as well as waist circumference [15].

However, chia seeds also expose the consumer to other elements, some of them potentially toxic elements (PTEs), such as Zn, Cd, or Al. This may mainly occur because of the environmental contamination of the soils in which chia is grown. In this regard, the ONE Health perspective reminds us of the interaction between environmental health and food safety and human health. In terms of regulation, it should be remembered that the European Union Commission Regulation (EU) 2023/915 on maximum levels for certain contaminants in certain foods only considers Pb, Cd, As, and Hg, and does not regulate other PTEs. Likewise, chia seeds are not covered by this regulation [16].

The existing studies tend to focus on the physiological effects of chia rather than evaluating whether it is a significant source of metals or are either very limited or somewhat outdated. To increase the knowledge on chia seeds and to respond to the growing interest in their nutritional and toxicological characterization, this study conducted a comprehensive exposure assessment along with a nutritional and toxicological evaluation. The study focused on determining the presence of both essential elements (sodium (Na), potassium (K), calcium (Ca), magnesium (Mg), iron (Fe), zinc (Zn), chromium (Cr), copper (Cu), molybdenum (Mo), manganese (Mn), and cobalt (Co)) and potentially toxic elements (PTEs), namely: nickel (Ni), strontium (Sr), barium (Ba), aluminum (Al), cadmium (Cd), lead (Pb), boron (B), and vanadium (V) in chia seeds. This analysis was conducted to assess the nutritional benefits and potential health risks associated with chia seed consumption.

## 2. Materials and Methods

### 2.1. Samples, Analytical Procedure, and Statistical Analysis

A sum of 20 samples of organic and conventional chia seeds of non-EU (60%) and EU (40%) origin were purchased through different distribution channels and analyzed. The protein content was assessed by analyzing sample labeling (range: 16 to 24%).

Five grams of each of the chia seed samples were weighed in triplicate in a porcelain capsule (Staatlich, Berlin, Germany) and dehydrated in an oven (Nabertherm, Lilienthal, Germany) at 80 °C for 24 h. Once dried, the capsule was weighed again and placed in a muffle furnace (Nabertherm, Lilienthal, Germany) for incineration. The temperature was gradually increased over 48 h until 450 ± 25 °C was reached and maintained for an additional 24 h [17]. The ashes obtained were exposed to acid digestion by adding 65% nitric acid (HNO_3_) (Sigma Aldrich, Taufkirchen, Germany) before being placed again into the muffle furnace for 24 h. In this case, the temperature was increased to 450 ± 25 °C for 12 h [18]. The final grayish-white ashes were finally dissolved in 1.5% HNO_3_ (Sigma Aldrich, Taufkirchen, Germany) to a volume of 25 mL [19]. The determination of the different elements (essential and potentially toxic) was carried out by inductively coupled plasma-optical emission (ICP-OES) using an autosampler Thermo Scientific iCAP PRO ICP-OES (Waltham, MA, USA). For quality control, reference materials were used for each of the elements. The recovery percentages (RPs) together with the limits of detection (LODs), limits of quantification (LOQs), and wavelength of each of the elements analyzed are listed in Table 1 along with the other operational parameters of the ICP-OES.

The statistical analysis of the data was performed using the GraphPad Prism 8.1.1 (GraphPad, San Diego, CA, USA) program for Windows. The Anderson–Darling, D’Agostino and Pearson, Shapiro–Wilk, and Kolmogarov–Smirnov tests were used to study the normality of the data [20]. To establish the existence or not of statistically significant differences (*p* < 0.05), the non-parametric Mann–Whitney test was used for data that did not follow a normal distribution, and the unpaired *t*-test for metals that did (K, Ca, Cu, and Zn) [21].

### 2.2. Exposure Assessment and Nutritional and Toxicological Characterization

Considering a mean adult body weight (b.w.) of 70 kg, the estimated daily intake (EDI) of each element was determined for two different consumption scenarios (Equation (1)). The first scenario considers a moderate daily consumption of 15 g of chia seeds, as this is the dosage recommended by most manufacturers. As several authors have reported positive health effects when higher daily intakes of chia seeds are consumed [22,23], a second consumption scenario (50 g/day) was considered.

Equation (1). Estimated daily intake (EDI):

*EDI* = *Element concentration* (mg/kg) × *Daily chia seed intake* (g/day) (1)

The EDIs were used for the nutritional and toxicological characterization by the percentage of contribution (Equation (2)). For the nutritional characterization of the essential elements (Na, K, Ca, Mg, Fe, Zn, Cr, Cu, Mo, Mn, and Co), the different gender nutritional reference intake (NRI) established by the Spanish Food Safety Agency (AESAN) were used as a reference value (Table 2). In the case of the PTEs (Ni, Sr, Ba, Al, Cd, Pb, B, and V), for the toxicological characterization, the Tolerable Daily Intake (TDI), Tolerable Weekly Intake (TWI), Tolerable Upper Intake Level (UL), and Benchmark Dose Lower Bound (BMDL10) established by the European Food Safety Authority (EFSA) and the Institute of Medicine (IOM) were used (Table 2). In the case of the PTEs, no gender approach was possible since the reference values are general.

Equation (2). Nutritional and toxicological characterization:

(2) $$Contribution\;of\;the\;EDI\;to\;the\;reference\;value\;\left(\%\right)=\frac{EDI}{\left(NRI/TDI/TWI/UL\right)}\times 100$$

For those essential elements and PTEs for which a reference dose (RfD) has been established (Table 3), a risk assessment was performed by calculating the Targeted Hazard Quotient (THQ) (Equation (3)) which is the quotient between the dose to which the consumer of these chia seeds is exposed and the RfD established for each of the elements mentioned in Table 3 [36,37,38,39]. If the Hazard Index (HI) (Equation (4)) ≥ 1, there is a moderate or serious risk to the health of the consumer from these quantities of seeds [40,41,42].

Equation (3). THQ [36,37,38]:

(3) $$THQ=\frac{Exposure\;dose}{RfD}=\frac{\frac{E_{f}\;\times \;C_{metal}\;\times \;D_{i}\;\times \;E_{d}}{B_{w}\;\times \;A_{t}}\;\times \;10^{-3}}{RfD}$$

- *E_f_*: exposure frequency (365 days/year).
- *C_metal_*: average concentration of each metal in chia seeds (mg/kg).
- *D_i_*: daily intake of PS (15 and 50 g/day).
- *E_d_*: average duration of exposure to chia seeds (25 years).
- *B_w_*: average weight (70 kg b.w.).
- *A_t_*: average exposure time (*E_f_* × *E_d_*).

Equation (4). HI [40,41,42]:

HI = ∑ THQ(4)

## 3. Results and Discussion

### 3.1. Essential Elements and PTEs in Chia Seeds According to the Production

Table 4 shows the concentrations of the essential elements and PTEs determined in the analyzed chia seeds depending on the type of production: conventional or organic.

**Figure 1:**
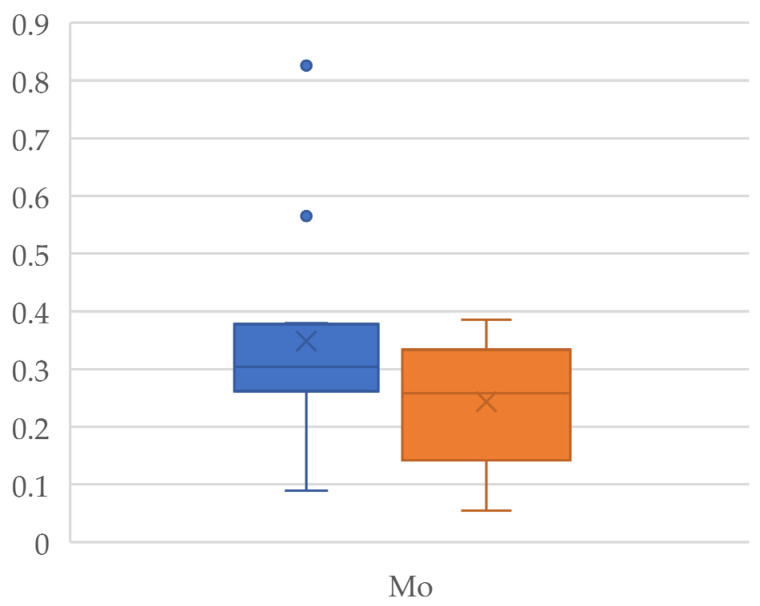
Box plot of the Mo concentrations detected in the organic (orange) and conventional (blue) chia seeds.

**Figure 2:**
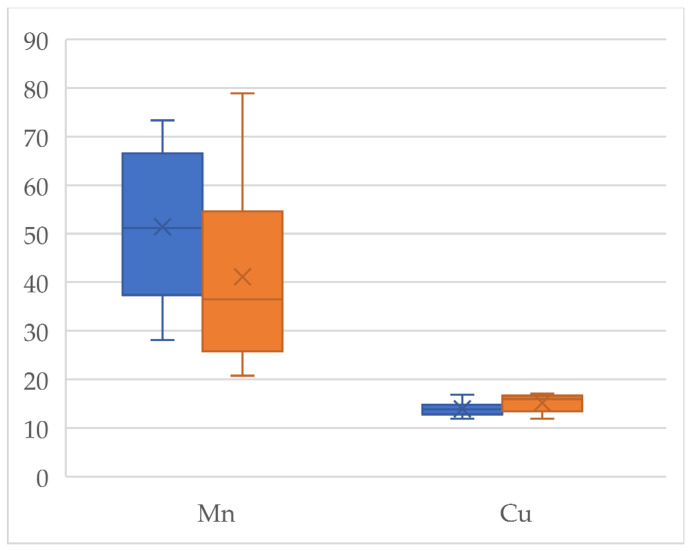
Box plot of the Mn and Cu concentrations detected in the organic (orange) and conventional (blue) chia seeds.

**Figure 3:**
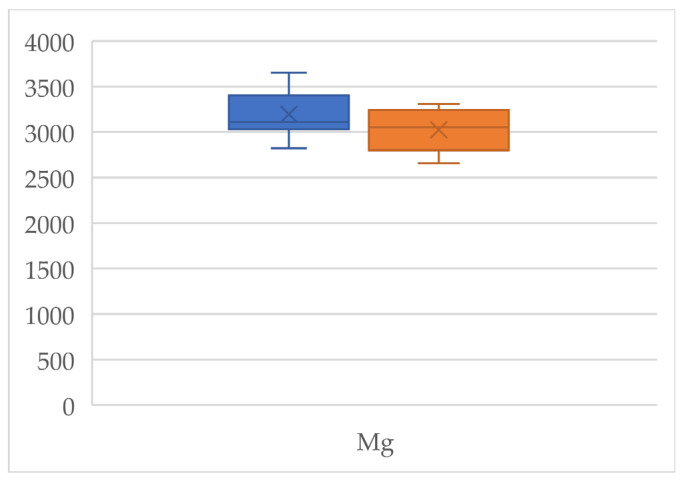
Box plot of the Mg concentrations detected in the organic (orange) and conventional (blue) chia seeds.

An organic product is understood to be one that has been obtained free from harmful chemicals to the environment during cultivation, i.e., it is environmentally friendly. For this reason, organic foods attract the attention of a large part of the population who associate the term “organic” with “natural” [43,44]. Organically produced chia seeds show higher levels of essential elements than conventional seeds, except for Na, K, and Zn. These novel products are considered a good source of Ca, and the results here show that this element reached 5811 mg/kg and 5726 mg/kg in organic and conventional chia seeds, respectively, with statistically significant differences between the two types of production (*p* = 0.01) [45]. The Ca levels found in chia seeds are higher than those found in milk, even though milk is considered by the population to be the main source of this mineral [14,46]. The differences observed between the levels of the different essential elements according to the type of production are statistically significant (*p* < 0.05) for Mo (*p* = 0.04) (Figure 1), Mn (*p* = 0.02), Cu (*p* = 0.01) (Figure 2), and Mg (*p* = 0.048) (Figure 3). Regarding Cu and Mn, the observed levels are higher in conventional seeds; this may be due to the use of certain chemical products containing these metals. No statistically significant differences were found for Co (*p* = 0.67), Cr (*p* = 0.08), Fe (*p* = 0.71), Ca (*p* = 0.43), K (*p* = 0.49), and Na (*p* = 0.65), so the levels of these metals are maintained regardless of the type of production.

In the case of PTEs, none of the analyzed samples showed detectable Pb levels, and organic chia seeds had higher concentrations of these elements, except for B. It is worth mentioning that the only chia seeds with V levels above the limit of quantification were also organic samples. Despite the different concentrations determined between the organic and conventional seeds, no statistically significant differences were found for Al (*p* = 0.47), Zn (*p* = 0.26), Ni (*p* = 0.71), B (*p* = 0.48), Sr (*p* = 0.87), and Ba (*p* = 0.57). Statistical analysis could not be carried out for V and Cd due to the small sample size.

Table 5 compares the levels of metals determined by the present study with previous studies reported in the last five years.

If we compare the levels detected now with those reported by the authors’ research group in 2018, it is easy to conclude that the occurrence of the elements, except Cr and Ba, is higher nowadays, with a striking increase in the levels of some essential elements including Na, Mg, K, Ca, and Mn [47]. As for PTEs, the difference in levels is not as noticeable as for the previously mentioned elements. In the case of Pb, only da Silva et al. detected levels in their samples (0.60 mg/kg). The highest Cd levels were detected by USDA (1.1 mg/kg) [48] and for Al (13.2 mg/kg) and B (11.2 mg/kg), the highest concentrations were observed by da Silva et al. [11].

It should be added that in a recent study conducted in Argentina in 2021 [49], where only the concentration of lead, cadmium, and arsenic in chia seeds was determined, concentrations similar to those of the aforementioned studies were obtained, especially to that of the study conducted in Brazil by da Silva et al [11].

### 3.2. Exposure Assessment

The labeling of some of the samples analyzed states that the daily intake of chia seeds should not exceed 15 g. If consumers follow these recommendations established by the industry, the EDIs of the different elements analyzed would be those shown in Table 5. The EDIs obtained are unlikely to pose a health risk regardless of the kind of production considered.

When considering the essential elements, as expected, those elements that are found in greater quantities are not those that contribute the most to the NRIs. The highest contribution percentage to the NRI was estimated for Mn, which was higher in the organic seeds (25.71%) than in the conventional seeds (20.56%), followed by Cu. In the case of Co, the daily intake of a portion of chia seeds contributes almost 2% of the Co TDI set by EFSA. Although the consumers of organic chia seeds will be exposed to higher amounts of Ni (0.024 mg/day), Sr (0.480 mg/day), and Ba (0.034 mg/day), these EDIs represent less than 5% of the reference intake values except for Sr.

The results show that the amounts of essential elements provided by the consumption of 15 g/day of chia seeds are nutritionally relevant since the contributions of the different EDIs to the reference intakes are estimated to be around 20% for elements such as Mn, Cu, and Mg. Nevertheless, some potential hazards regarding Cu, Mn, Cr, and Mg were identified when consuming 50 g/day of conventional chia seeds since the EDI from this individual foodstuff would reach 58.26% and 68.85% of the Cu NRI set for men and women, respectively; 85.70% of the NRI set for Mn if organic chia seeds are consumed; and 40% of the NRI set for Cr and Mg in women. Given these results, the authors conclude that because chia seeds provide considerable amounts of these essential elements, consumers following rich diets in chia seeds may be at risk of exceeding the NRIs established for these elements if total diet dietary exposure assessments were conducted [24].

In the case of the PTEs, a dose of 50 g/day of chia seeds is a relevant source of Sr (17% of the TDI). As these high consumers may also be exposed to Sr from drinking water and other foods such as cereals, dairy products, seafood, and green leafy vegetables, the risk of exceeding the TDI (0.13 mg/kg b.w./day) established by the IOM [11,25,48] cannot be underrated. For the rest of the PTEs, it is unlikely that the consumption of these foods poses a health risk as the EDIs are well below 5% of the reference intakes except for Ni which exceeds 8% (Table 6). In the case that the reference dose was TWI, it was mathematically transformed into TDI for the calculation of the contribution percentage.

The risk associated with the chronic consumption of chia seeds was also evaluated using the THQ and HI approach for individuals of 70 kg b.w., the two consumption scenarios previously described (15 g and 50 g of conventional or organic chia seeds/day), and a timeline of 25 years (Table 7).

For both types of seeds (organic and conventional), while the intake of the recommended dosage by the manufacturers (15 g/day) does not pose a health risk, the daily consumption of 50 g of chia seeds may suggest a health risk as HI > 1.

Co is the element that contributes most to the HI in both types of production. In the case of conventional seeds, Co is closely followed by Cu (0.274) while in organic seeds it is Mn (0.262). Interestingly, Co was not the element that contributed the most to the reference intake via EDI, as shown in Table 5, but rather Cu and Mn, which are in second position in the THQ risk evaluation. V contributes to a lesser extent to the HI for organic seeds (0.006) and conventional seeds (0.004). In general, as expected, the PTEs are the ones that contribute the least to the THQ calculation.

The limitations of this study are that the chia seeds analyzed may not be representative in general terms for the entire population. Although chia seeds have been selected from a multitude of origins, studies with chia seeds in other continents could be necessary.

## 4. Conclusions

Chia seed consumption has surged in recent years, driven by perceived health benefits. While rich in essential nutrients, chia seeds also contain PTEs. Despite the differences between organic and conventional production, our study reveals that these two types of production exhibit comparable levels of both essential nutrients and PTEs.

Consumers following the moderate dosage recommendations specified by most manufacturers (15 g/day) are not expected to be exposed to high intakes of PTEs or health risks. However, the population following a daily chia seed-rich diet (50 g/day) will be intaking more than half of the Cu and Mn daily requirements just from this food group and, therefore, are at risk of exceeding the nutritional reference intakes if all the dietary sources are considered.

Since Commission Regulation (EU) 2023/915 of 25 April 2023 on maximum levels for certain contaminants in food does not consider these novel foods or some of the PTEs detected in the present study, it would be advisable to promote its monitoring, risk analysis, and regulation.

## Figures and Tables

**Table 1:** Operational parameters and quality control of the method.

RF Power		1150 W			Nebulizer Gas Pressure		0.2 L/min	
Nebulizer Gas Flow		12.5 L/min			Auxiliary Gas Flow		0.5 L/min	
Cool gas flow		12.5 L/min			Pump speed		45 rpm	
**Element**	**Emission Wavelengths (nm)**	**LOD (mg/L)**	**LOQ (mg/L)**	**Reference Material**		**Concentration Recorded (mg/kg)**	**Concentration Certified (mg/kg)**	**RP (%)**
**Al**	167.0	0.005	0.015	SRM 1515 Apple Leaves		286 ± 9	285.10 ± 26	99.7
**Sr**	407.7	0.003	0.011			25.00 ± 2.00	24.60 ± 4.00	98.3
**Cr**	267.7	0.001	0.005			0.29 ± 0.03	0.30 ± 0.00	97.8
**Co**	228.6	0.001	0.005			0.090 ± 0.00	0.09 ± 0.00	100
**Mo**	202.0	0.0016	0.005			0.090 ± 0.01	0.09 ± 0.02	99.4
**B**	249.6	0.008	0.027			27.00 ± 2.00	27.00 ± 1.50	99.9
**Pb**	214.4	0.0009	0.003			0.456 ± 0.054	0.47 ± 0.024	94.8
**Cd**	214.4	0.0007	0.002	SRM 1573 a Tomato Leaves		1.40 ± 0.070	1.52 ± 0.04	92.3
**Na**	818.3	2.221	7.404	SRM 1548 a Typical Diet		8132 ± 942	8001.90 ± 4.76	98.4
**Ca**	315.8	1.629	5.432			1967 ± 113	161.10 ± 158	99.7
**K**	766.4	1.764	5.883			6970 ± 125	6858.50 ± 318	98.4
**Mg**	383.8	1.580	5.268			580 ± 26.70	575 ± 25.70	98.1
**Ni**	221.6	0.0009	0.003			0.37 ± 0.02	0.38 ± 0.04	102.3
**Ba**	455.4	0.0006	0.002			1.10 ± 0.10	1.13 ± 0.09	102.5
**Zn**	213.8	0.0027	0.009	SRM 1567 a Wheat Flour		11.60 ± 0.40	11.40 ± 0.20	98.2
**Mn**	257.6	0.0008	0.003			9.40 ± 0.90	9.30 ± 0.50	98.9
**Fe**	238.2	0.004	0.013			14.10 ± 0.50	13.90 ± 0.30	98.9
**V**	292.4	0.0014	0.004			0.011 ± 0.00	0.011 ± 0.00	99.4
**Cu**	324.7	0.003	0.011			2.10 ± 0.20	2.09 ± 0.40	99.7

**Table 2:** Nutritional and toxicological reference values of the different elements.

	Element	Gender	Reference Value			Element	Gender	Reference Value	
	Nutritional Reference Intake (NRI) [24,25]					Tolerable Daily Intake (TDI)			
**Essential elements**	Na	M/F	1500	mg/day	**PTEs**	Ni	M/F	13 μg/kg b.w./day [26]	
	K	M/F	3500	mg/day		Sr	M/F	0.13 mg/kg b.w./day [27]	
	Ca	M/F	950	mg/day		Ba	M/F	0.2 mg/kg b.w./day [28]	
	Mg	M	350	mg/day		**Tolerable Weekly Intake (TWI)**			
		F	300	mg/day		Al	M/F	1 mg/kg b.w./week [29]	
	Fe	M	9.1	mg/day		Cd	M/F	2.5 mg/kg b.w./week [30]	
		F 20–59 years	18	mg/day		**Benchmark Dose Lower Bound (BMDL_10_)**			
		F ≥ 60 years	9	mg/day		Pb cardiotoxicity	M/F	1.5	μg/kg b.w./day [31]
	Zn	M	11	mg/day		Pb nephrotoxicidad		0.63	
		F	8	mg/day					
	Cr	M	35	μg/day		**Tolerable Upper Intake Level (UL)**			
		F	25	μg/day		B	M/F	0.16 mg/kg b.w./day [32]	
	Cu	M	1.3	mg/day		V	M/F	0.026 mg/kg b.w./day [33]	
		F	1.1	mg/day					
	Mo	M/F	65	μg/day					
	Mn	M/F	3	mg/day					
	**Tolerable Daily Intake (TDI)** [34]								
	Co	M/F	0.0016	mg/kg b.w./day					

NRI: the amount of nutrients required for the proper functioning of the body depending on the population under consideration and its physiological conditions [25]. TDI: the amount of substance present in different food sources that can be consumed daily throughout the whole day without posing a risk to health [35]. TWI: the amount of substance present in different food sources that can be consumed weekly throughout the week without risk to health [35]. UL: maximum daily intake of a substance from different dietary sources to which an individual can be exposed daily over a lifetime without risk to health [35]. BMDL_10_: the lowest dose of a substance that results in a low health risk [35]. M: male; F: female.

**Table 3:** Reference dose for PTEs and essential elements [39].

Metal	Reference Dose (RfD) (mg/kg/day)
**Mn**	0.14
**Cu**	4 × 10^−2^
**Zn**	0.3
**Fe**	0.7
**Cr**	3 × 10^−3^
**Mo**	5 × 10^−3^
**Ni**	2 × 10^−2^
**Co**	3 × 10^−4^
**V**	5.04 × 10^−3^
**Ba**	0.07
**Sr**	0.6
**Al**	4 × 10^−4^
**B**	0.2
**Cd**	1 × 10^−3^
**Pb**	1 × 10^−7^

**Table 4:** Levels of metals in chia seeds according to their type of production.

	Element (mg/kg)	Organic Chia Seeds				Conventional Chia Seeds			
		Mean Concentration ± SD	Min. Value	Max. Value	N (>LQ)	Mean Concentration ± SD	Min. Value	Max. Value	N (>LQ)
**Essential elements**	**Na**	182 ± 44.56	110	244	12	194 ± 32	156	248	8
	**K**	5458 ± 501	4583	6502	12	5543 ± 423	4933	6315	8
	**Ca**	5811 ± 531	4941	6706	12	5726 ± 250	5274	6041	8
	**Mg**	3195 ± 270	2821	3650	12	3025 ± 234	2656	3308	8
	**Fe**	51.74 ± 9.75	43.69	78.55	12	50.72 ± 6.03	43.38	62.16	8
	**Zn**	44.27 ± 3.67	44.27	51.99	12	45.54 ± 6.03	37.56	58.03	8
	**Cu**	13.92 ± 1.33	11.95	16.83	12	15.15 ± 1.90	11.89	17.05	8
	**Mo**	0.35 ± 0.19	0.09	0.83	12	0.24 ± 0.11	0.05	0.39	8
	**Mn**	51.42 ± 16.34	28.09	73.34	12	41.13 ± 19.42	20.76	78.93	8
	**Co**	0.16 ± 0.12	0.05	0.48	12	0.13 ± 0.06	0.06	0.22	8
	**Cr**	0.23 ± 0.07	0.16	0.43	12	0.19 ± 0.02	0.16	0.23	8
**PTEs**	**Al**	8.79 ± 6.92	2.87	21.66	12	9.65 ± 9.23	2.14	30.76	8
	**Cd**	0.01 ± 0.00	<LQ	0.01	1	0.03 ± 0.00	<LQ	0.03	1
	**Pb**	<LQ	<LQ	<LQ	0	<LQ	<LQ	<LQ	0
	**Ni**	1.57 ± 0.61	1.06	2.75	12	1.46 ± 0.46	0.84	2.07	8
	**Sr**	32.01 ± 4.69	27.40	44.04	12	31.25 ± 2.60	27.95	34.76	8
	**Ba**	2.28 ± 0.51	1.75	3.23	12	2.15 ± 0.42	1.51	2.71	8
	**B**	6.17 ± 0.78	5.24	7.69	12	6.27 ± 0.76	5.15	7.52	8
	**V**	0.04 ± 0.02	<LQ	0.07	5	0.03 ± 0.00	<LQ	0.03	1

N (>LQ): number of samples above LQ.

**Table 5:** Comparison of essential element and PTEs levels in chia seeds.

Element (mg/kg)		Rubio et al. [47]	da Silva et al. [11] *		USDA [48]	The Present Study
**Essential elements**	**Na**	70	1500	160	1400	187
	**K**	2853	6200	4070	5500	5492
	**Ca**	2556	4800	6310	4300	5777
	**Mg**	1709	3500	3350	3300	3127
	**Fe**	44.60	93.90	77.20	76.90	51.33
	**Zn**	32.6	36.50	45.8	3.76	44.78
	**Cu**	11.30	13.20	9.24	6.30	14.41
	**Mo**	0.28	-	2.00	-	0.31
	**Mn**	26.10	40.50	27.23	24.80	47.30
	**Co**	0.14	-	-	-	0.15
**PTEs**	**Al**	4.49	13.2	-	9.90	9.13
	**Cd**	0.01	0.8	-	1.1	0.02
	**Pb**	<LD	0.60	-	0.00	<LQ
	**Ni**	0.74	-	-	-	1.53
	**Sr**	10.09	-	-	-	31.71
	**Ba**	3.26	-	-	-	2.33
	**B**	5.86	11.2	-	9.3	6.21
	**V**	<LD	-	-	-	0.04
	**Cr**	0.28	0.00	-	0.00	0.21
	**Li**	1.81	-	-	-	2.71

* mineral composition in chia seeds grown in different places: Rio Grande do Sul (RS) and Mato Grosso (MT), Brazil.

**Table 6:** Estimated daily intakes (EDI) and contributions to reference intake values when consuming 15 g and 50 g of organic and conventional chia seeds/day.

Element		Organic Chia Seeds						Element		Conventional Chia Seeds				
		C_mean_ (mg/kg)	Gender Age	Consumption Scenarios						C_mean_ (mg/kg)	Consumption Scenarios			
				15 g/day		50 g/day					15 g/day		50 g/day	
				EDI (mg/day)	% NRI	EDI (mg/day)	% NRI				EDI (mg/day)	% NRI	EDI (mg/day)	% NRI
**Essential elements**	**Na**	182	M/F	2.74	0.18	9.12	0.61	**Na**	194		2.91	0.19	9.69	0.65
	**K**	5458	M/F	81.87	2.34	272.88	7.80	**K**	5543		83.15	2.38	277.16	7.92
	**Mg**	3195	M	47.92	13.69	159.74	45.64	**Mg**	3025		45.38	12.97	151.26	43.22
			F		15.98		53.25					15.13		50.42
	**Ca**	5811	M/F	87.16	9.18	290.53	30.58	**Ca**	5726		85.89	9.04	286.29	30.14
	**Mo**	0.35	M/F	3.65 × 10^−3^	8.04	1.74 × 10^−2^	26.80	**Mo**	0.24		3.65 × 10^−3^	5.61	1.22 × 10^−2^	18.70
	**Mn**	51.42	M/F	0.77	25.71	2.57	85.70	**Mn**	41.13		0.62	20.56	2.06	68.55
	**Cu**	13.92	M	0.21	16.07	0.70	53.55	**Cu**	15.15		0.23	17.48	0.76	58.26
			F		18.99		63.29					20.66		68.85
	**Fe**	51.74	M	0.78	8.53	2.59	28.43	**Fe**	50.72		0.76	8.36	2.54	27.87
			F 29–59 years		4.31		14.37					4.23		14.09
			F ≥ 60 years		8.62		28.74					8.45		28.18
	**Zn**	44.27	M	0.67	6.04	2.21	20.12	**Zn**	45.54		0.68	6.21	2.28	20.70
			F		8.30		27.67					8.54		28.46
	**Cr**	0.23	M	3.41 × 10^−3^	9.75	1.14 × 10^−3^	32.49	**Cr**	0.19		2.87 × 10^−3^	8.20	9.56 × 10^−3^	27.32
			F		13.65		45.49					11.47		38.25
	**Element**	**C_mean_ (mg/kg)**	**Gender**	**EDI (mg/day)**	**% TDI**	**EDI (mg/day)**	**% TDI**	**Element**	**C_mean_ (mg/kg)**		**EDI (mg/day)**	**% TDI**	**EDI (mg/day)**	**% TDI**
	**Co**	0.16	M/F	2.36 × 10^−3^	2.11	7.9 × 10^−3^	7.02	**Co**	0.13		1.91 × 10^−3^	1.71	6.37 × 10^−3^	5.69
**PTEs**	**Element**	**C_mean_ (mg/kg)**	**Gender**	**EDI (mg/day)**	**% TDI**	**EDI (mg/day)**	**% TDI**	**Element**	**C_mean_ (mg/kg)**		**EDI (mg/day)**	**% TDI**	**EDI (mg/day)**	**% TDI**
	**Al**	8.79	M/F	0.13	1.32	0.44	4.39	**Al**	9.65		0.15	1.45	0.48	4.83
	**Cd**	0.01	M/F	2.22 × 10^−4^	0.89	7.41 × 10^−4^	2.97	**Cd**	0.03		4.83 × 10^−4^	1.93	1.61 × 10^−3^	6.43
	**Ni**	1.57	M/F	0.02	2.59	0.08	8.63	**Ni**	1.46		0.02	2.41	0.07	8.03
	**Sr**	32.01	M/F	0.48	5.28	1.60	17.59	**Sr**	31.25		0.47	5.15	1.56	17.17
	**Ba**	2.28	M/F	0.03	0.24	0.11	0.81	**Ba**	2.15		0.03	0.23	0.11	0.77
	**Element**	**C_mean_ (mg/kg)**	**Gender**	**EDI (mg/day)**	**% UL**	**EDI (mg/day)**	**% UL**	**Element**	**C_mean_ (mg/kg)**		**EDI (mg/day)**	**% UL**	**EDI (mg/day)**	**% UL**
	**B**	6.17	M/F	0.09	0.83	0.31	2.76	**B**	6.27		0.09	0.84	0.31	2.80
	**V**	0.04	M/F	6.4 × 10^−4^	0.04	2.12 × 10^−3^	0.12	**V**	0.03		4.45 × 10^−4^	0.02	1.48 × 10^−3^	0.08

M: male; F: female.

**Table 7:** Mean values of the exposure dose, TQH and HI.

Elements	Organic Chia Seeds				Conventional Chia Seeds			
	Daily Intake				Daily Intake			
	15 g/day		50 g/day		15 g/day		50 g/day	
	Exposure Dose (mg/kg/day)	THQ	Exposure Dose (mg/kg/day)	THQ	Exposure Dose (mg/kg/day)	THQ	Exposure Dose (mg/kg/day)	THQ
**Mn**	1.10 × 10^−2^	0.079	3.67 × 10^−2^	0.262	8.81 × 10^−3^	0.063	2.94 × 10^−2^	0.210
**Cu**	2.98 × 10^−3^	0.075	9.95 × 10^−3^	0.249	3.25 × 10^−3^	0.081	1.08 × 10^−2^	0.270
**Zn**	9.49 × 10^−3^	0.032	3.16 × 10^−2^	0.105	9.76 × 10^−3^	0.033	3.25 × 10^−2^	0.108
**Fe**	1.11 × 10^−2^	0.016	3.70 × 10^−2^	0.053	1.09 × 10^−2^	0.016	3.62 × 10^−2^	0.052
**Cr**	4.87 × 10^−5^	0.016	1.62 × 10^−4^	0.054	4.10 × 10^−5^	0.014	1.37 × 10^−4^	0.046
**Mo**	7.47 × 10^−5^	0.015	2.49 × 10^−4^	0.050	5.21 × 10^−5^	0.010	1.74 × 10^−4^	0.035
**Ni**	3.37 × 10^−4^	0.017	1.12 × 10^−3^	0.056	3.13 × 10^−4^	0.016	1.04 × 10^−3^	0.052
**Co**	3.37 × 10^−5^	0.112	1.12 × 10^−4^	0.374	2.73 × 10^−5^	0.091	9.10 × 10^−5^	0.303
**B**	1.32 × 10^−3^	0.007	4.41 × 10^−3^	0.022	1.34 × 10^−3^	0.007	4.48 × 10^−3^	0.023
**V**	9.09 × 10^−6^	0.002	3.03 × 10^−5^	0.006	6.36 × 10^−6^	0.001	2.12 × 10^−5^	0.004
**Cd**	6.89 × 10^−6^	0.007	2.30 × 10^−5^	0.023	3.18 × 10^−6^	0.003	1.06 × 10^−2^	0.011
**Sr**	6.86 × 10^−3^	0.011	2.29 × 10^−2^	0.038	6.70 × 10^−3^	0.011	2.23 × 10^−2^	0.037
**Ba**	4.89 × 10^−4^	0.007	1.63 × 10^−3^	0.023	4.60 × 10^−4^	0.007	1.53 × 10^−3^	0.022
**HI**	-	**0.391**	**-**	**1.304**	-	**0.356**	**-**	**1.185**

## Data Availability

The original contributions presented in this study are included in the article. Further inquiries can be directed to the corresponding author(s).
